# Economic Assessment of FMDv Releases from the National Bio and Agro Defense Facility

**DOI:** 10.1371/journal.pone.0129134

**Published:** 2015-06-26

**Authors:** Dustin L. Pendell, Thomas L. Marsh, Keith H. Coble, Jayson L. Lusk, Sara C. Szmania

**Affiliations:** 1 Department of Agricultural Economics, Kansas State University, Manhattan, Kansas, United States of America; 2 School of Economic Sciences and Paul G. Allen School for Global Animal Health, Washington State University, Pullman, Washington, United States of America; 3 Department of Agricultural Economics, Mississippi State University, Starkville, Mississippi, United States of America; 4 Department of Agricultural Economics, Oklahoma State University, Stillwater, Oklahoma, United States of America; 5 Signature Science, LLC, Austin, Texas, United States of America; University of Liverpool, UNITED KINGDOM

## Abstract

This study evaluates the economic consequences of hypothetical foot-and-mouth disease releases from the future National Bio and Agro Defense Facility in Manhattan, Kansas. Using an economic framework that estimates the impacts to agricultural firms and consumers, quantifies costs to non-agricultural activities in the epidemiologically impacted region, and assesses costs of response to the government, we find the distribution of economic impacts to be very significant. Furthermore, agricultural firms and consumers bear most of the impacts followed by the government and the regional non-agricultural firms.

## Introduction

Scientific laboratories designed to study diseases are not completely risk free, and the possibility exists that accidents of nature or deliberate acts of terror might cause the spread of a disease that the facility is trying to prevent. On the one hand animal and human health officials in the United States are interested in preventing the introduction and spread of diseases like Ebola, Rift Valley Fever or Highly Pathogenic Avian Influenza, while on the other hand scientists within the United States need small quantities of these materials for study and to develop response strategies should an outbreak occur. It is a tenuous balancing act with tradeoffs that can be staggering varying in outcomes and economic value. This study considers a timely and relevant case, food-and-mouth-disease (FMD), in one such facility, the National Bio and Agro Defense Facility (NBAF). We couple outcome from plume and epidemiological models with an economic model to calculate the potential economic consequences of several critical types of accidental releases from a research facility. The approaches used here are likely to have application for other diseases and research facilities, and in providing a monetary estimate of the risks posed by such facilities that can be compared against the benefits of better preparedness. In 2009, Manhattan, Kansas was selected as the site for the new National Bio and Agro Defense Facility. It is intended to replace the current research facility at Plum Island Animal Disease Center (PIADC) in New York, the primary animal disease research facility since 1954, to enhance research and education. The FY 2010 DHS Appropriation Act (P.L. 111–83), however, would not allow funds to be made available for construction of NBAF until:

“…a site-specific bio-safety and bio-security mitigation risk assessment, which includes an integrated set of analyses using plume modeling and epidemiologic impact modeling…, and the results of the National Academy of Sciences’ review of the risk assessment…”[1].

Economic analysis is congressionally mandated as part of the site specific risk assessment, necessary to link outcomes from the plume and epidemiological models to risk outcomes. A focal point of the risk assessment centers on potential accidental releases of viruses from NBAF and the subsequent consequences of such releases. Releases of viruses from research facilities are not necessarily common, but have uncertain economic consequences and have occurred in the past[2].

The purpose of the current study is to estimate the economic consequences associated with unintentional, hypothetical releases of foot-and-mouth disease virus (FMDv) from NBAF. Specifically, we assess the economic consequences to agricultural firms and consumers, quantify costs and disruptions to non-agricultural activities in the epidemiologically impacted region, and assess costs of response to the government. Different from previous economic studies of FMD outbreaks in the United States [3–6] and across the world [7], the current study is unique as it examines unintentional aerosol, liquid waste, transference, and tornado releases from an animal research facility. This set of releases provides a nearly complete coverage of the feasible risk space, providing a broad landscape over which release events could translate into a range of economic consequences. Given the inherent uncertainty in an FMD outbreak, economic consequences are assessed over a distribution of epidemiological outcomes. Finally, the extensive and intensive nature of this site specific risk assessment (e.g., plume, epidemiological, socioeconomic data, information, and modeling) over a large study region is unprecedented in previous FMD studies focused on the U.S.

The findings of this study have important implications from modeling to policy. First, the distribution of economic consequences between unannounced (aerosol, liquid waste, and transference) and announced (tornados) releases are differentiated. While the releases are hypothetical, they are plausible as demonstrated by recent, similar FMDv events occurring across the world[2]. Second, a multi-step risk assessment evolved whereby an updated site specific risk assessment followed the initial site specific risk assessment. A purpose of the updated risk assessment is to respond to limitations identified by both the National Academy of Sciences and public comments. Third, mitigation control includes stamping out, vaccinate-to-live, and vaccinate-to-kill. A concern is that in the event of large outbreaks killing all infected animals may not be feasible. Fourth, uncertainty is carried from the plume model through the epidemiologic model outcomes to the economic assessment, identifying distributions of potential economic outcomes and not a simple point estimate of economic consequences. Finally, intertemporal welfare effects are differentiated across agricultural firms and commodities from production to feeding, processing, and down the supply chain to consumption. As a result, we are able to examine the impacts of FMDv releases over time and across commodities along the supply chain.

## Background

There is an urgent need to develop better surveillance, diagnostics, response, and recovery to contagious diseases. Between 1940 and 2004, 60% (or 335) of emerging infectious diseases in the United States were zoonotic, a disease that is transmissible between animals and humans, with 72% originating from wildlife [8]. Trends suggest that the frequency of emerging infectious diseases will continue to increase, especially with the growing interface between humans, animals, and wildlife [9].

Potential transmission and spread of foreign animal diseases (FAD) and zoonotic diseases are driven by some of the same forces that propel the global economy. Globalization has increased both international trade and human mobility. As the world human population and standard of living continues to grow, there is, and will be a continued increase in demand for protein from livestock. The United Nations World Tourism Organization [10] forecasts a 3 to 4% increase over 2012s record one billion international tourist arrivals worldwide in 2013.

The economic losses associated with emerging, highly contagious FADs and zoonotic diseases can be significant. According to the World Bank [11], the direct costs (e.g., costs to public and animal health services and producer compensation for culled animals) and indirect costs (e.g., trade and tourism) of outbreaks during the 2000s surpassed $20 billion and $200 billion, respectively. Hosono et al. [12] estimated that the 1998–1999 Nipah Virus outbreak in Malaysia resulted in $90 million in losses from culled animals and $170 million in indirect effects. The 2003 severe acute respiratory syndrome (SARS) outbreak in East Asia and Canada resulted in estimated losses of $40 to $50 billion [11]. In the 2001 FMD outbreak in the UK that lasted more than 220 days in duration, over six million animals were culled with estimated losses of $10 billion to $12 billion [13].

Animal agriculture is important to the U.S. economy as it is a primary source of food and nutrition, a major contributor to exports, and it is valued at $165 billion [14]. With such a vital industry, the United States needs to position itself to defend against the threat of FADs and zoonotic diseases. To do so, a modern biocontainment laboratory that is capable of conducting research and developing vaccines against such diseases is required, yet the United States does not currently have one. In 2004, in the *Homeland Security Presidential Directive 9 (HSPD-9)*: *Defense of United States Agriculture and Food*, the Administration showed the need for such a facility:

“The Secretaries of Agriculture and Homeland Security will develop a plan to provide safe, secure, and state-of-the-art agriculture biocontainment laboratories that research and develop diagnostic capabilities for foreign animal and zoonotic diseases” [15].

There are 14 foot-and-mouth disease virus incidents known or believed to have been released over the past 50 years from biocontainment research laboratories worldwide, including Czechoslovakia, Demark, Germany, Spain, Russia, United Kingdom, and United States [2]. Because of potential release and subsequent consequences of a contagious infectious disease, proper analyses and reviews must be conducted before constructing a new state-of-the-art biocontainment research facility.

## The National Bio and Agro-Defense Facility

Research on FADs, such as FMDv, is conducted in biocontainment research laboratories throughout the world, including the Canadian Science Centre for Human and Animal Health (Winnipeg, Canada), Australian Animal Health Laboratory (Geelong, Australia), the Institute for Animal Health (Pirbright, United Kingdom), and the Friedrich-Loeffler-Institute (Riems, Germany). In the United States, Plum Island Animal Disease Center (PIADC) in New York has been the livestock disease research facility since 1954. However, the Department of Homeland Security [16] has noted that PIADC is at “the end of its lifecycle and is too small to meet the nation's research needs”.

It is the intent that NBAF will: (1) replace and enhance the current research at PIADC; (2) enhance research capabilities diagnosing foreign animal, emerging and zoonotic diseases in large animal livestock; (2) develop new vaccines and other countermeasures for large animal livestock; (3) train veterinarians and animal agricultural specialists in preparing for and responding to animal diseases; and (4) give the United States its only BSL-4 research capacity of high-consequence diseases affecting large animal livestock. Because of two Government Accountability Office [2, 17] reports, the FY 2010 DHS Appropriation Act (P.L. 111–83) would not allow funds to be made available for construction of NBAF until it completed a site specific risk assessment to be reviewed by the National Academies of Sciences [1].

Following the Appropriations Act of 2010, DHS commissioned the *Site-Specific Biosafety and Biosecurity Mitigation Risk Assessment* in 2010, including a review by the National Academy of Sciences (NAS). In the NAS review, they noted several shortcomings in the site specific risk assessment (SSRA), including an inadequate qualitative risk assessment, underestimation of the risk of a pathogen release and transmissions, and methodological flaws of the plume and epidemiological modeling [18]. To address these shortcomings, DHS conducted an *Updated Site-Specific Biosafety and Biosecurity Mitigation Risk Assessment* in 2012 [16], which was also reviewed by the National Academy of Sciences [19]. The information from initial risk assessment completed in 2010 was used in altering the design of NBAF and changing the standard operating procedures, personnel training, and emergency response planning. For example, the initial design of NBAF would withstand wind speeds of 90 miles per hour [20]. Following the updated risk assessment, the design of NBAF was modified to conform to nuclear regulatory standards and withstand with up to 228 miles per hour [16].

## Modeling Framework

To assess the economic impacts of unintentional FMDv releases from NBAF, we follow [4, 7, 21–22] to link supply shocks from an animal disease spread model with a multi-commodity, multi-market partial equilibrium model. This is supplemented with a regional input-output economic model to capture impacts on allied and associated businesses [4, 21]. Additional economic shocks include domestic and international markets, which are discussed in detail below. Government costs associated with controlling and eradicating and FMD outbreak are calculated as well.

### Epidemiological Disease Spread Model

The epidemiological disease spread model used in this study is the North American Animal Disease Spread Model (NAADSM). NAADSM is a spatial, stochastic, state-transition simulation model that simulates highly contagious animal diseases [23]. NAADSM has been used in numerous studies to evaluate the impacts of highly contagious animal diseases in various countries including several economic impact studies [3–4, 6, 21–22]. The NAADSM framework requires extensive parameterization including information on animal population (e.g., location, production type, size of herd), disease manifestation (e.g., latently infected), disease transmission (e.g., direct, indirect, and aerosol), disease detection, surveillance, and control (e.g., animal movements, traceability, and vaccination). The parameters can take on an integer value (e.g., number of herds destroyed per day), probability (e.g., probability of infection given exposure to an infected herd), probability density function (e.g., length of time that an infected herd is subclinically infectious) or relational function (e.g., probability of detecting an infectious herd, which is a function of clinical signs being observed and being reported to authorities once clinical signs have been observed). The parameters are developed through literature searches, research, and expert opinions. Given the epidemiological output is exogenous input into the economic model, and not the primary focus of this paper, the reader is referred to DHS [16, section 6.1.4] for complete documentation of the parameter values used in this study.

NAADSM is simulated 200 times for each scenario to generate a distribution of disease spread outcomes. In addition to the uncertainty resulting from the stochastic disease spread model, different starting locations for infection are also incorporated to better reflect uncertainty. Unlike previous studies which pick a specific starting location (e.g., cow-calf operation or feedlot operation) to model the consequences of an FMD outbreak, this study includes all possible starting locations (e.g., cow-calf operations, feedlot operations, etc.) for each release scenario. In other words, for each starting location the FMD outbreak is modeled and the consequences are ranked. It is then possible to report the outbreak consequences at the probability levels (e.g., P5, P50, and P95) for the different starting locations.

### Economic Model

#### Agricultural Firms and Consumers

A quarterly multi-market partial equilibrium simulation model is used to assess how unintentional releases of FMDv would impact U.S. agricultural producers and consumers. This study utilizes an updated version of the Paarlberg et al. [22] partial equilibrium model. The partial equilibrium model includes major agricultural sectors along vertical and horizontal market chains beginning with livestock and grain production to meat processing and the final consumer, including both domestic and international. Exogenous, production, domestic demand, and international trade, shocks resulting from an FMD outbreak are incorporated into the model as percentage changes from the baseline. The economic model then solves for the percent changes in the endogenous variables (prices and quantities) for each quarter. The percent changes in the endogenous variables are then applied to a baseline defined by the observed data for the first quarter of 2009 through the fourth quarter of 2018 of no-disease. This results in estimated changes in per capita consumer welfare and changes in quasi-profits and captured by returns to capital and management.

Parameters for the partial equilibrium model include livestock-feed balance information, revenue and factor shares, and elasticities. The livestock-feed balance information, revenue shares, and factor shares are retained as defined in [22]. The retail elasticity values for final meat demand for beef, pork, and poultry [24], lamb [25], and milk [26] are updated for this study. Substitution elasticities for derived demand and trade elasticities remained unchanged. Producer expectations regarding expected future returns are modeled as naïve expectations [27]. Livestock supply, use, and price data, as well as forage prices are from the Livestock Marketing Information Center [28]. Coarse grains, wheat, rice, and the soybean complex supply, use, and price data are from Outlook Reports and Data prepared by the USDA-Economic Research Service [29]. Total quarterly use was generated by feed balance equations from which data on animal numbers are combined with standard feeding practices to produce quarterly amounts of forage and pasture. Hay, corn silage, and sorghum silage production is reported by the National Agricultural Statistics [30]. Uncut grazed pasture is imputed for quarters 2 and 3. International trade data are derived from LMIC [28], USDA-ERS [31], and Foreign Agricultural Service [32]. Information concerning the crop policy mechanisms are from Provisions of the Federal Agriculture Improvement and Reform Act of 1996 [33] and the 2002 Farm Act [34].

#### Regional Non-Agricultural Sector

Assessing the costs and disruptions to the non-agricultural activities in the epidemiologically impacted region can be very important [4, 21]. Thompson et al. [13] estimates that the direct losses of tourism following the 2001 UK FMD outbreak were equal to the losses to the agricultural sector, excluding the producer compensation from the government. Furthermore, the indirect effects to tourism were more than 20 times larger when compared to the indirect effects to agriculture. The regional impacts in this study are estimated using an input-output model which is a system of equations that describe the flow of income and product throughout an economy. Specifically, the Bureau of Economic Analysis’s Regional Input-Output Modeling System (RIMSII) is used because they provide a well-accepted and validated methodology to evaluate these impacts. Moreover, the input-output industrial multipliers provide the flexibility to define the states defined by the disease spread model.

RIMSII integrates the input and output relationships of approximately 500 U.S. industries and regional economic accounts. The final-demand multipliers for output are used to estimate the indirect economic activity generated by a specific economic activity in a region. Thus, the intent of using the RIMSII data is to measure the effects of an FMD outbreak on the non-agricultural regional economy. Calculations are structured to remove duplication or double counting of losses. The indirect effects evaluated include: (1) the effect of culling and destroying animals on the non-agricultural regional economy (e.g., retail trade); (2) the economic implication of a travel ban that would limit recreational and non-essential travel in and out of a region; and (3) the indirect effects from the stimulus to the region created by the expenditures during government eradication and clean-up efforts. Travel bans, resulting in reduced tourism, are another important source of potential economic losses for the impacted region [35]. Travel bans composed of transit and ground transportation; spectator sports; hotels and motels; and food and drink services are evaluated.

The economic impact from the loss in travel expenditures can be measured using RIMSII [36]. Total domestic travel expenditures for overnight trips and day trips of over 50 miles in 2007 were obtained from the U.S. Statistical abstract produced by the U.S. Census Bureau by state. However, the RIMSII data separates the economic effects of various forms of travel (Table 1). Thus, using data on the percentage allocations of travel expenditures from the Bureau of Labor Statistics, expenditures are allocated by category for each state in the study region [37].

**Table 1:** Allocation of Travel Expenditure by Category (%).

Category		%
	Air Transportation	29.10
	Transit and Ground Passenger Transportation	13.95
	Spectator Sports	10.80
	Hotels and Motels, Including Casino Hotels	19.08
	Food Service	27.08

While not insignificant, travel and tourism is not a dominant sector in the region. Kansas, Nebraska, and Oklahoma each constitute less than 1% of the U.S. domestic travel visits and expenditures (3.1% combined). Travel and tourism are more important in Texas, Colorado and Missouri which contribute 6.7%, 2.0% and 1.8% of domestic travel visits and expenditures, respectively. Travel expenditures, by state, for trips of over 50 miles were obtained from the U.S. Census Bureau. Additionally, the U.S. Bureau of Labor Statistics reports the percentage of allocations of travel expenditures. Thus, using data on the percentage allocations of travel expenditures, expenditures were allocated by category (e.g., air transportation) for each state in the study region ([16]). It appears likely that in major outbreaks travel restrictions to non-agricultural events will be lifted after two quarters so a maximum reduction of 4% of annual travel is realized. For outbreaks of less than two quarters, the travel reduction is computed from the number of days the outbreak lasts as a percentage of a full year’s reduction.

### Government Costs

Typical government costs associated with eradicating an FMD outbreak are calculated. These costs include appraisal, cleaning and disinfection, disposal, euthanasia, indemnification, quarantine, surveillance, and vaccination. Indemnification payments reflect the value of culled animals at average market prices in the first quarter of 2009 prior to an FMDv release. Table 2 reports the government costs used in this study which are based on published literature [38–39] and the non-indemnification costs per animal are consistent in magnitude with those reported by Abdalla et al. [40].

**Table 2:** Government Costs used in Mitigating and Eradicating an FMD Outbreak.

Cost Category	Cow-Calf	Dairy	Feedlot	Swine	Sheep
Cost of appraisal for slaughter ($/Herd)^a^	95.35	95.35	238.37	95.35	95.35	
Cost of cleaning and disinfection ($/herd)^a^	1,776.40	3,762.80	11,173.75	1,279.81	1,776.40	
Fixed costs of surveillance ($/herd)^b^	225.7	225.7	225.7	225.7	225.7	
Variable costs of surveillance ($/visit)^b^	84.64	84.64	112.85	84.64	84.64	
Quarantine costs($/animal/day)^b^	1.41	1.41	1.41	1.41	1.41	
Euthanasia ($/animal)^a^	27.91	5.7	5.79	27.91	4.1	
Carcass disposal ($/animal)^a^	14.97	2.24	2.08	2.89	14.97	
Fixed costs of vaccination ($/herd)^b^	338.55	564.25	902.8	654.25	338.55	
Variable costs of vaccination ($/animal)^b^	6.77	6.77	6.77	6.77	6.77	

Note: Assumed 28 day quarantine period with all susceptible premises in each state incurring quarantine. Assumed 3 surveillance visits per herd.

^a^ Inflated to 2009 dollars [39].

^b^ Inflated to 2009 dollars [38].

### Exogenous Shocks

An FMD outbreak would result in shocks to production, domestic demand, and international trade [5, 7, 22]. These shocks are expressed as percentage changes for each quarter and incorporated into the economic model.

#### Production (Supply) Shock

Output from the disease spread model is used to estimate the production or supply shocks. Specifically, the number of animals culled by production type, by quarter is used to calculate the percentage reduction in supply. Additionally, two emergency vaccinations scenarios are assumed: vaccinate-to-kill and vaccinate-to-live. No federal vaccination policy for FMD exists in the U.S. and no definitive precedent was uncovered in previous studies. After discussions with members of a U.S. government review panel, the vaccination-to-live and vaccination-to-kill policies were defined for this study as initial assessment. It is realized that different assumptions concerning a vaccination policy could be made (see [3]). For outbreaks <180 days, the vaccinate-to-kill scenario is assumed where all vaccinated animals are assumed to be culled. For outbreaks >180 days, it is assumed that culling of the vaccinated cattle will occur until the 180^th^ day of the outbreak and then afterwards, any vaccinated cattle in the queue to be culled or newly vaccinated cattle will not be culled and remain in the cattle inventory. Depending on the scenario (vaccinate-to-kill or vaccinate-to-live), the number of animals culled and/or animals vaccinated listed in Table 3 and Table 4 are used in calculating the production shock, respectively.

**Table 3:** Number of Animals Depopulated by Production Type and Duration of FMD Outbreak.

Event	Output/Location	Duration (days)	Vaccination*	Production Type				
				Beef Slaughter Cattle	Beef Cows	Dairy	Swine	Sheep
Liquid Waste	p5/p5	0	V2K	0	0	0	0	0
	p5/p50	0	V2K	0	0	0	0	0
	p5/p95	15	V2K	21,640	25	0	0	0
	p50/p5	78	V2K	1,093,786	5,667	6,507	105,646	608
	p50/p50	424	V2L	2,707,326	53,592	81,606	2,012,587	12,884
	p50/p95	473	V2L	5,864,177	140,795	399,528	6,376,951	119,768
	p95/p5	335	V2L	3,548,413	0	0	2,504,141	0
	p95/p50	492	V2L	10,333,905	276,457	807,009	14,758,579	73,298
	p95/p95	533	V2L	12,324,616	338,167	1,175,595	13,440,254	72,937
Aerosol	p5/p5	28	V2K	13,665	0	88	0	4,368
	p5/p50	48	V2K	44,905	1,038	678	16,463	4,511
	p5/p95	180	V2K	767,388	10,305	29,982	162,219	1,042
	p50/p5	83	V2K	537,320	3,789	1,452	53,757	435
	p50/p50	424	V2L	2,998,536	52,026	63,523	3,856,818	10,418
	p50/p95	473	V2L	7,943,054	289,095	552,631	10,131,995	66,806
	p95/p5	492	V2L	11,035,317	154,198	758,512	13,547,509	111,235
	p95/p50	492	V2L	10,357,307	276,457	837,208	14,633,068	73,298
	p95/p95	533	V2L	12,354,311	338,167	1,161,410	13,453,333	72,937
Transference	p5/p5	35	V2K	207,849	793	224	10,252	5
	p5/p50	48	V2K	283,757	838	224	11,910	5
	p5/p95	420	V2L	811,038	13,413	14,663	89,342	5,798
	p50/p5	266	V2L	3,417,986	0	0	2,596,428	0
	p50/p50	424	V2L	3,417,986	0	0	2,596,428	0
	p50/p95	473	V2L	4,322,953	60,553	158,820	2,207,841	10,731
	p95/p5	492	V2L	10,013,500	101,207	420,751	10,640,659	116,893
	p95/p50	492	V2L	11,082,943	154,198	675,692	13,799,764	111,235
	p95/p95	533	V2L	10,391,913	223,173	763,259	13,784,818	73,298
Tornado	p5/p5	240	V2L	3,421,445	0	0	2,547,784	0
	p5/p50	252	V2L	3,401,560	1,038	590	2,852,239	143
	p5/p95	252	V2L	3,797,229	6,240	10,249	2,905,886	5,719
	p50/p5	272	V2L	3,379,717	0	0	2,759,748	0
	p50/p50	424	V2L	3,755,606	147	504	2,599,759	0
	p50/p95	473	V2L	8,512,799	101,207	375,891	8,396,696	116,893
	p95/p5	333	V2L	3,582,124	1,158	0	2,416,037	0
	p95/p50	492	V2L	4,217,057	80,383	82,913	9,253,821	9,760
	p95/p95	533	V2L	9,574,132	238,228	713,783	10,055,838	12,974

*V2K is vaccinate-to-kill, V2L is vaccinate-to-live.

**Table 4:** Number of Animals Vaccinated by Production Type.

Event	Output/Location	Production Type				
		Beef Slaughter Cattle	Beef Cows	Dairy	Swine	Sheep
Liquid Waste	p5/p5	0	0	0	0	0
	p5/p50	0	0	0	0	0
	p5/p95	1,513	6,642	525	1,944	445
	p50/p5	558,237	572,587	30,573	701,383	32,083
	p50/p50	2,674,860	2,468,126	504,503	21,585,087	310,240
	p50/p95	3,830,901	10,242,637	877,355	26,840,569	1,166,088
	p95/p5	919,314	3,224,388	139,881	801,670	145,166
	p95/p50	8,682,751	20,620,026	1,714,854	29,677,167	1,936,414
	p95/p95	7,911,697	21,792,460	1,650,836	31,526,353	2,148,883
Aerosol	p5/p5	5,048	30,848	1,104	3,672	1,492
	p5/p50	115,651	134,906	25,423	731,283	9,009
	p5/p95	465,865	1,235,676	53,852	921,923	64,157
	p50/p5	177,582	364,830	16,911	451,631	24,521
	p50/p50	2,960,231	5,099,550	515,408	22,353,735	411,781
	p50/p95	4,941,577	14,733,683	875,965	25,788,110	1,148,413
	p95/p5	7,288,962	15,182,268	1,359,066	30,865,915	1,552,347
	p95/p50	8,317,797	20,559,373	1,650,610	29,879,112	1,923,440
	p95/p95	7,906,093	21,591,189	1,615,099	31,643,619	2,138,106
Transference	p5/p5	24,472	33,938	1,289	21,058	4,156
	p5/p50	141,976	86,811	3,089	150,330	6,392
	p5/p95	744,111	662,275	70,573	2,443,715	50,374
	p50/p5	692,486	2,573,193	48,822	568,146	110,748
	p50/p50	3,452,187	3,080,166	559,902	22,002,431	335,381
	p50/p95	5,494,909	15,502,989	1,033,836	25,232,099	1,425,357
	p95/p5	7,026,349	15,253,943	1,298,846	30,119,740	1,546,958
	p95/p50	8,236,661	18,135,449	1,599,610	27,766,499	1,806,057
	p95/p95	7,865,919	21,867,560	1,568,794	31,672,812	2,137,911
Tornado	p5/p5	581,040	2,563,305	50,015	826,824	109,144
	p5/p50	672,299	2,817,234	66,321	1,137,185	112,406
	p5/p95	1,118,966	3,164,052	96,510	2,704,629	140,056
	p50/p5	753,106	2,772,602	70,235	538,916	98,617
	p50/p50	912,234	4,253,702	73,533	961,011	166,595
	p50/p95	4,317,099	12,922,165	935,192	24,917,275	1,246,109
	p95/p5	866,156	3,418,977	73,107	1,137,993	142,008
	p95/p50	3,029,146	9,656,230	401,631	25,220,883	569,723
	p95/p95	5,261,933	16,096,332	1,278,449	26,985,036	1,476,384

#### Domestic Demand Shock

There are anticipated decreases in consumer demand, even though FMD does not pose a human health concern. As such, reduction in U.S. consumer demand is incorporated to allow variations in the level of consumer perception of food quality [41]. These domestic demand shocks represent the share of the U.S. population decreasing consumption of a final good and provide a policy instrument by which to manage impacts on final demand. Because there have been no FMD events on the U.S. mainland since 1929, it is necessary to draw information from other events both domestically and internationally. Various studies have quantified the impact of consumer demand in the United States to livestock and meat disease outbreaks [41–45]. These studies all have similar conclusions; the impacts are small and short lived, but the likelihood of larger responses coincided with larger outbreaks/recalls. Although FMD has not occurred in recent history on the U.S. mainland, there have been FMD outbreaks experienced in other countries, including the UK in 2001. Consumers in the UK decreased their average weekly per capita carcass meat consumption from 235 grams in 2001 to 229 grams in 2002 (or 2.7% decline). Consumption in the UK recovered to pre-outbreaks levels by 2006 [46].

Given the above information, domestic demand shocks are specified for FMD across the scenarios. Based on the epidemiological output (see Table 4) smaller (larger) outbreaks coincided with outbreaks that lasted shorter (longer) than one quarter. Consequently, following an FMD outbreak lasting less than one quarter, it was assumed that 5% of people would refrain from consuming beef, pork, and lamb while 2.5% would stop consuming milk and dairy products during the outbreak. This is consistent with consumer reactions to food safety events reported in [41, 44]. In the second quarter, domestic consumer demand for beef, pork and lamb declined by 2.5% and was fully recovered (i.e., 0% decline) for dairy and milk products. It is assumed that consumer demand for meat products would be fully recovered by the third quarter.

In outbreaks lasting more one quarter, it was assumed that 10% of domestic consumers would refrain from consuming beef, pork and lamb while 5% would stop consuming milk and dairy products during the outbreak. Following the end of the outbreak, it was assumed that domestic consumer demand would decrease by 5% for one quarter and 2.5% for another quarter for beef, pork, and lamb. Consumer demand for dairy and milk products would decline by 2.5% for one quarter following the outbreak.

#### International Trade Shock

The magnitude and duration of trade shocks assumed in this study are based on observations from previous events in throughout the world, including the United States. In 2003 and 2004, due to isolated incidences of BSE, the U.S. and Canada faced complete bans on beef in major overseas markets while beef and cattle imports and exports continued among the North America Free Trade Agreement countries (Canada, Mexico, and the United States) under a variety of restrictions [47]. The United States has experienced a long recovery relative to pre-outbreak trade status as a result the isolated BSE events. U.S. beef exports, as a percentage of beef production was 9.5% in 2003, dropped dramatically to 1.9% in 2004, and recovered to 7.4% in 2008 [28].

A review of previous literature is useful in identifying plausible time lengths defining trade bans for our FMD scenarios. The EU imposed a one year ban on the UK following its 2001 FMD outbreak. Rich and Winter-Nelson [7] analyzed the 2000–2001 FMD outbreaks in the southern cone of South America and concluded short lived impacts on exports to Argentina, Brazil, and Uruguay. Randolph, Morrison, and Poulton [48] assumed a 12 month ban on exports during FMD outbreaks in Zimbabwe. Nogueira et al. [49] and Tozer et al. [50] apply 1 to 2 year trade bans for hypothetical FMD outbreaks in Mexico and Australia, respectively. Although the actual length of export restrictions will depend upon the actual product, disease, trade agreements, and countries involved, these observations provide valuable information on trade bans.

Given the information above, trade shocks are created in the following manner. First, 95% of all U.S. exports of beef, pork, lamb meat, cattle, swine, and sheep are halted during the full quarter of the outbreak and for one quarter after the last case appears. This assumes some processed/cooked beef is still exported after the outbreak. The interruption of exports for one quarter beyond the end of the outbreak (and for two quarters beyond the end of the outbreak when emergency vaccination without slaughter is practiced) is consistent with World Organization for Animal Health (OIE) guidelines and practices (Chapter 8.5) during FMD outbreaks [51]. Second, after the additional quarter ended with no FMD reported, it is assumed that gradual recovery of U.S. exports will occur until it reaches the baseline levels. Full recovery is assumed to occur in approximately two years following one full quarter after the outbreak is eradicated. For FMD, the duration of the outbreak becomes a critical element in determining the economic effects from trade disruptions.

### Study Region

The region of focus for this study includes seven states: Colorado, Iowa, Kansas, Missouri, Nebraska, Oklahoma and Texas. In this region agriculture is economically important, especially for livestock. In 2009, cattle and calves are the most valuable agricultural commodity in four states in the study [52]. All seven states are in the top 10 cattle and calves inventory with 45.1 million head (47.7% of total U.S. inventory) located in those seven states. Texas, Nebraska, Kansas, Iowa, and Colorado are the five largest states with cattle on feed; almost 10 million head on January 1, 2009 [52]. Furthermore, hogs are recognized as one of the top five commodities those states. Total hog and pig inventory on December 1, 2008 for the region was 30.8 million head (47.4% of total U.S. inventory). 31.2 percent of total U.S. sheep and lamb inventory (1.8 million head) occurred in those seven states on January 1, 2009 [52]. Additionally, a significant percentage of state farm receipts in this region are derived from dairy.

A unique feature of this study when compared with previous work is the size of the livestock population and number of herds. Most hypothetical FMD studies in the United States focus on a small region at the state or county level [4, 6, 27, 38, 53–54]. With nearly 83.76 million head and 0.45 million herds, this study contains one of the largest, if not the largest, number of susceptible animals/herds of any FMD economic modeling study (Table 5). The exact farm locations are not available in the United States. Location data typically exists for medium and large sized operations. Smaller operations were accounted for by incorporating locations from a dataset developed by Lawrence Livermore National Laboratory (LLNL) using the NASS Agricultural Census data from 2007 [55].The production types used in NAADSM are adjusted to allow for use in the partial equilibrium economic model. The production types required by the partial equilibrium economic model are beef cattle, dairy, slaughter cattle, swine and sheep. As such, the production types used in NAADSM (listed in Table 5) are adjusted as follows: Cow-Calf + Beef (backyard) = beef cattle; Dairy = dairy; Feedlot (small) + Feedlot (large) = beef slaughter; swine = Swine (small) + Swine (large) + Swine (backyard); Goats + Sheep + Small ruminants (backyard) = sheep.

**Table 5:** Number of Animals and Herds by Production Type in Study Region.

Production Type^1^		Animals	Herds
*Cattle*			
	Cow-calf	25,443,443	243,661
	Dairy	2,377,253	5,565
	Feedlot (small)	4,856,559	22,514
	Feedlot (large)	11,465,365	1,797
	Beef (backyard)	542,927	72,544
	**Total Cattle**	44,685,547	346,081
*Swine*			
	Swine (small)	437,402	15,824
	Swine (large)	34,487,716	11,818
	Swine (backyard)	497,914	9,873
	**Total Swine**	35,423,032	37,515
	*Small Ruminant*		
	Goats	1,476,917	32,327
	Sheep	2,065,225	13,518
	Small ruminants (backyard)	113,263	19,273
	**Total Small Ruminant**	3,655,405	65,118

^1^ The production types required by the partial equilibrium economic model are beef cattle, slaughter cattle, dairy, swine and sheep. The production types used in NAADSM and listed in this Table are adjusted as follows: Cow-Calf + Beef (backyard) = beef cattle; Feedlot (small) + Feedlot (large) = beef slaughter; Dairy = dairy; Swine (small) + Swine (large) + Swine (backyard) = swine; Goats + Sheep + Small ruminants (backyard) = sheep.

### Release Events

As with any contagious disease research laboratory, there are multiple mechanisms and pathways in which a pathogen might be released from a containment laboratory. Such pathways include: liquid (e.g., through a drain), solid waste (e.g., carcass disposal system), fomite and vectors (e.g., clothing, mosquito), and aerosol (e.g., air filtration system). Although protocols to reduce the risk of infectious material leaving the laboratory exist, it is impossible to eliminate all of the risk associated when dealing with highly contagious pathogen research. Thus, four plausible unintentional introduction events of the FMDv are evaluated. These events with examples of actual releases include:

1. Liquid Waste Release
  In August 2007, an FMD outbreak occurred near Pribright, UK. According to DEFRA [56], it has been suggested that the probable cause of the outbreak was due to leaking drainage pipes at the Institute for Animal Health, a zoonotic disease research laboratory.
2. Aerosol Release
  In September 1971, an air leak in a gasket around a research laboratory door was the cause of in an internal release of FMDv at PIADC [2]. This scenario represents the virus accidentally released through an aerosol.
3. Transference Release
  In August 1980, several steers at PIADC were found to contain a different strain of FMDv than the vaccine research being conducted. Although the actual cause of this outbreak was never determined, it is likely a laboratory worker carried and transmitted the virus to steers [2].
4. Tornado Release
  Although no know natural disaster has resulted in a release of FMDv, it is plausible that such an event could occur as 42 tornados, on average, are reported with 120 nautical miles of NBAF each year [16]. On June 11, 2008, an Enhanced Fujita 4 tornado touched down in Manhattan, Kansas, including on the campus of Kansas State University, which is where NBAF will be located.

Each of the four release scenarios have differing: (1) probabilities of an event occurring, (2) amount of FMDv released, and (3) the means by which the virus is transported in the environment. Thus, this information is used to calculate the probability that any given premises becomes infected with FMDv. Further details about the different release mechanisms can be found in [16] pages 405–412 and 480–507.

Additionally, the first three events represent *unannounced* events while the latter scenario represents an *announced* event. It is important to distinguish between these two types of unintentional releases as an unannounced release could continue to spread the FMDv until the disease is identified and confirmed by officials. In the event of an announced released, control and mitigation plans (e.g., animal movement bans, increased surveillance, etc.) could be immediately implemented, potentially reducing the impacts.

## Results

### Epidemiological Output

The results from the disease spread model are summarized in Tables 3 and 4. Results are reported for the distribution of outcomes at 5^th^, 50^th^, and 95^th^ percentiles of outbreaks based on animals culled. This provides a range of consequences that may arise from an FMD outbreak. To better reflect uncertainty inherent in the potential different outbreak starting locations (i.e., cow-calf operation, feedlot, etc.), additional consequences are reported for the 5^th^, 50^th^, and 95^th^ percentiles based on location of the index case. For example, P5/P50 implies the 5^th^ percentile of epidemiological output based on culled animals and the 50^th^ percentile of epidemiological output based on starting location.

In all release events, there were several assumptions regarding vaccinations including: (1) an emergency vaccination program was implemented after the first animal was infected; (2) the FMD vaccination supply was not a limiting constraint; (3) a 10 km vaccination ring was employed; (4) all animals that were vaccinated were culled unless the outbreak lasted longer than 2 quarters, at which the culling of vaccinated animals ceased at the end of the 2^nd^ quarter. Those animals that were vaccinated and not culled remained in the inventory.

In the aerosol, liquid waste, and transference release events, there are 10 scenarios in which the outbreak lasted less than 2 quarters (i.e., vaccinate-to-kill policy); 7 of those scenarios were less than 49 days in duration (Table 3). Additionally, the number of animals culled in those scenarios generally was less than 1.2 million head. The remaining scenarios were a vaccinate-to-live policy and ranged in duration from 266 to 533 days. The number of animals culled ranged between 6.0 and 27.4 million head for the longer duration scenarios.

The tornado release event results have one noticeable difference: the upper bound of animals culled and duration of outbreaks was smaller when compared to the other three release events, but the lower bound was much higher. In other words, the 5^th^ percentile of epidemiological output resulted in 6.0 million head culled with a duration of 240 days, which is much larger than the other three release events (Table 3). The number of animals culled and length of the outbreak for the 95^th^ percentile of epidemiological output scenario was 20.6 million head and 533 days. Although the duration of 533 days was the same as other three release event scenarios, the number of animals culled was much smaller in the 95^th^ percentile scenario. The impacts at the 5^th^ percentile were larger due to the initial spread of the virus by the tornado. Because this release event is in effect a self-announcement, mitigation and control plans are put into place immediately and a higher probability of observing and reporting the disease by a producer, which can be seen at the 95^th^ percentile of epidemiological output based on culled animals.

The numbers of livestock vaccinated follow a similar pattern as duration and number of animals culled. The P5/P5 and P95/P95 scenarios result in the smallest and largest number of vaccinations administered, respectively (Table 4). The fewest vaccines administered are 0 in the Liquid Waste release event for P5/P5 while the P95/P95 scenario for the Transference release scenario results in over 65 million vaccinations.

### Economic Consequences

To determine the total economic impact for a scenario, the changes in producer returns to capital and management and consumer welfare, government indemnification and non-indemnification expenditures, and the costs to the non-agricultural regional sector were summed together.

#### Liquid Waste Release

Losses for the liquid waste release scenario range from $0 to $138,889 million in damage (Table 6). The 5^th^ percentile epidemiological output and 5^th^ and 50^th^ location quartiles resulted in no detection and spread of FMD (Table 4). Thus, no economic damages were incurred. In the P5/P95 scenario (5^th^ percentile in epidemiological output and 95^th^ percentile for starting location), changes in producer returns to capital and management were a decline of $19,920 million while changes in consumer welfare increased by $3,489 million. The positive effect on consumers was a result of a small production shock, small adverse reactions from consumers, and trade sanctions. In other words, it is possible for consumers of meat and dairy products to benefit from an FMD outbreak, if it is small in nature and consumer reactions are limited because of trade sanctions on agricultural exports leads to oversupply and reduce U.S. meat and dairy prices [3, 22, 49–50]. Producer and consumer impacts for P50/P5 are similar in size to P5/P95 while the remaining distribution of epidemiological and starting location impacts range from declines of $47,578 to $64,027 million and declines of $43,436 to $60,322 million for producers and consumers, respectively. In all release event scenarios, the negative impacts to consumers are smaller than that of the producers.

**Table 6:** Agricultural and Regional Non-Agricultural Impacts and Government Costs of Hypothetical FMD Outbreak (Millions $).

		Changes in		Government Costs			
Event	Output/Location	Producer Returns to Capital and Management	Consumer Surplus	Indemnification	Non-Indemnification	Regional Non-Agriculture Impacts	Total
Liquid Waste	p5/p5	$0	$0	$0	$0	$0	$0
	p5/p50	$0	$0	$0	$0	$0	$0
	p5/p95	-$19,920	$3,489	$18	$3	-$39	-$16,491
	p50/p5	-$18,752	$4,061	$1,414	$288	-$1,143	-$17,536
	p50/p50	-$56,869	-$51,990	$1,903	$1,730	-$3,726	-$116,218
	p50/p95	-$64,027	-$60,322	$4,614	$3,216	-$4,152	-$136,331
	p95/p5	-$47,578	-$43,436	$2,322	$771	-$831	-$94,938
	p95/p50	-$61,231	-$58,915	$8,377	$5,387	-$2,433	-$136,343
	p95/p95	-$60,679	-$58,701	$9,844	$5,604	-$4,061	-$138,889
Aerosol	p5/p5	-$19,909	$3,480	$30	$6	-$50	-$16,515
	p5/p50	-$19,733	$3,483	$259	$103	-$179	-$16,791
	p5/p95	-$28,154	-$26,128	$1,594	$373	-$2,827	-$59,076
	p50/p5	-$19,349	$3,697	$691	$154	-$438	-$16,935
	p50/p50	-$56,573	-$51,877	$2,187	$2,097	-$2,440	-$115,174
	p50/p95	-$62,610	-$60,274	$6,424	$3,984	-$2,600	-$135,892
	p95/p5	-$60,842	-$59,389	$8,693	$4,954	-$3,063	-$136,941
	p95/p50	-$61,238	-$58,965	$8,416	$5,367	-$3,718	-$137,704
	p95/p95	-$60,646	-$58,723	$9,850	$5,600	-$4,645	-$139,464
Transference	p5/p5	-$19,796	$3,576	$163	$27	-$163	-$16,573
	p5/p50	-$19,660	$3,646	$320	$62	-$310	-$16,706
	p5/p95	-$58,229	-$52,239	$521	$270	-$1,982	-$113,241
	p50/p5	-$39,473	-$33,066	$2,252	$702	-$983	-$76,476
	p50/p50	-$55,919	-$51,213	$3,107	$2,348	-$1,627	-$114,214
	p50/p95	-$61,445	-$59,895	$7,457	$4,241	-$4,087	-$137,125
	p95/p5	-$60,726	-$59,506	$8,662	$4,925	-$3,062	-$136,881
	p95/p50	-$61,238	-$59,170	$8,269	$5,028	-$3,128	-$136,833
	p95/p95	-$60,544	-$58,966	$9,850	$5,621	-$3,543	-$138,524
Tornado	p5/p5	-$39,496	-$33,174	$2,252	$706	-$802	-$76,430
	p5/p50	-$39,560	-$33,108	$2,264	$763	-$1,176	-$76,871
	p5/p95	-$39,262	-$33,009	$2,522	$918	-$3,131	-$78,842
	p50/p5	-$39,478	-$33,220	$2,243	$725	-$760	-$76,426
	p50/p50	-$56,074	-$52,452	$2,585	$1,228	-$2,622	-$114,961
	p50/p95	-$62,412	-$60,102	$6,327	$3,670	-$4,147	-$136,658
	p95/p5	-$47,557	-$43,605	$2,350	$786	-$2,204	-$96,502
	p95/p50	-$64,450	-$61,619	$3,396	$3,041	-$2,281	-$134,787
	p95/p95	-$62,056	-$59,981	$7,410	$4,290	-$3,828	-$137,565

The regional non-agricultural effects were negative and range from $0 to losses of $4,152 billion (Table 6). Although government indemnification payments replace the value of lost livestock, it is not enough to offset the full economic impact on the region. The government costs associated in eradicating an FMD outbreak range from $0 to $15,448 million with 50 to 85 percent of that due to indemnity payments (Table 6).

**Fig 1:**
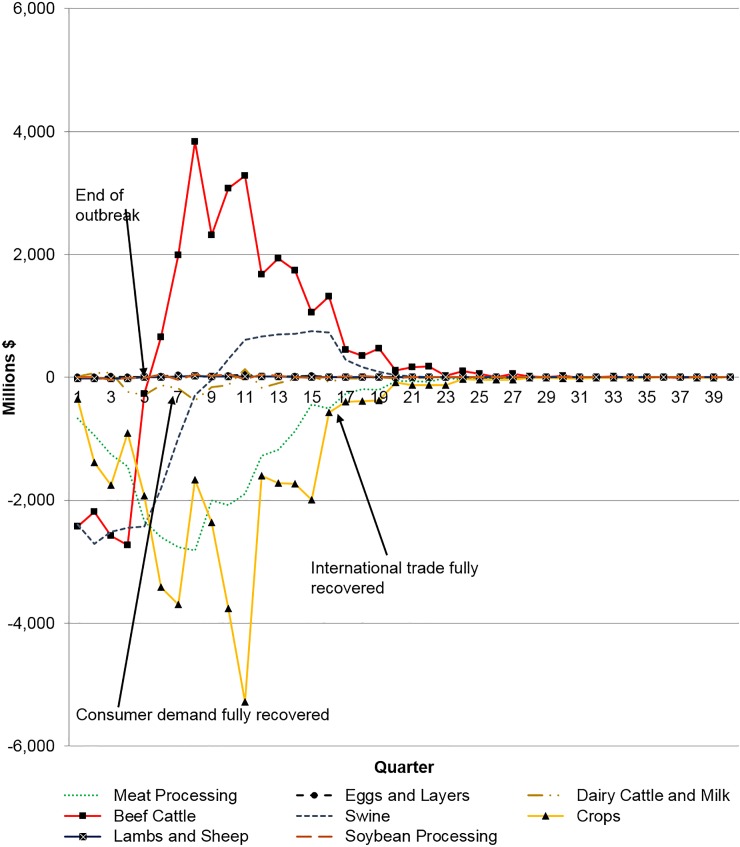
Changes in Producers Returns to Capital and Management for Liquid Waste Event, P50/P50 Scenario.

Table 6 summarizes the distributional cumulative economic impact across the entire study period for the liquid waste release (beginning in 2009 and ending in 2018). However, consequences of disease outbreaks are inherently dynamic in nature with benefits and costs accruing differently to producers and consumers across time, and this interplay has important policy implications [5]. Fig 1 illustrates the changes in producer returns to capital and management by agricultural sector across time for the P50/P50 Liquid Waste scenario. After an outbreak is announced there is an immediate negative effect on the swine and beef cattle sectors of about $2,400 million. This is due to the loss of livestock, meat, and dairy export markets, reduced demand for red meat and dairy products by domestic consumers, and culled animals. When the outbreak is officially declared over (the 5^th^ quarter), the beef cattle sector’s producers returns to capital and management has rebounded to the pre-outbreak levels while the swine sector’s recovery to pre-disease outbreak levels occurs approximately four quarters later (the 9^th^ quarter). This result is mostly due to the amount of exports lost by both sectors; approximately 7% and 17% of U.S. beef and swine production in 2009 was exported, respectively. After the 5^th^ quarter, beef producers experience positive returns, and do so for the next 17 quarters, primarily a result of lower grain and forage prices, consumer demand fully recovering, and the gradual recovery of export markets. Swine producers also have positive gains, but not as large of gains as the beef cattle producers.

Losses to the meat processing sector’s returns to capital and management decline from the onset of the outbreak until the peak in the 8^th^ quarter, where the loss in that quarter is approximately $2,813 billion (Fig 1). These losses are a result of lower beef prices due to the oversupply of beef (i.e., loss of the export markets). Similar to the meat processing, producer returns to management and capital in the crops sector decline. However, the losses in the crops sector are more severe and continue to decline until the 11^th^ quarter. These losses (which include grains, forage, and pasture) are a result of less demand from fewer cattle and swine. Additionally, the 2009 grains prices were far above U.S. government support levels, thus, price support payments had little, if any, effect. Both the meat processing and crops sectors producer returns do not fully recover to pre-disease levels by the 40^th^ quarter. Although producer returns to capital and management for the remaining sectors (eggs and layers, dairy cattle and milk, lambs and sheep, and soybean processing) are positive or negative, they are very small.

Table 7 reports aggregate changes in producer returns to capital and management across the 40 quarters by sector. This perhaps gives the best overview of the impacts to the agriculture industry in this hypothetical FMD outbreak. Additional Liquid Waste scenarios reported in DHS [16] remain qualitatively similar, but do vary according to the degree of the outbreak.

**Table 7:** Changes in Aggregate Producer Returns to Capital and Management for Liquid Waste Event, P50/P50 Scenario.

Sector	Millions $
Meat Processing	-26,055.61
Eggs and Layers	248.97
Dairy Cattle and Milk	-1,634.26
Beef Cattle	14,696.28
Swine	-10,577.89
Lambs and Sheep	72.86
Crops	-35,989.54
Soybean Processing	187.59

#### Aerosol Release

In the event of an accidental release of the FMDv through an aerosol, the total economic impacts range from losses of $16,515 million to $139,464 million (Table 6). The range of losses to the agricultural producers was $19,733 million for the P5/P50 scenario to $62,610 million for the P50/P95 scenario. The small localized FMD outbreaks that lasted less than a quarter in duration, P5/P5, P5/P50, and P5/P50 scenarios, resulted in gains to consumers of about $3,500 million. The P5/P95 scenario resulted in a decline in consumer welfare of $26,128 million while the remaining scenarios saw much larger declines in consumer welfare ranging from $51,877 to $60,274 million because of the duration of the outbreak and number of animals culled and vaccinated. The government indemnification costs range from $30 million for the P5/P5 scenario to $9,850 million for the P95/P95 scenario. The non-indemnification costs are smaller with a range of $6 million to $5,600 million. Finally, the impacts to the regional non-agricultural sectors are declines of $50 million to $4,645 million across the scenarios. The distribution of losses by production type across time is qualitatively similar to the Liquid Waste scenario described above.

#### Transference Release

Because the duration of the FMD outbreaks and number of animals culled and vaccinated are similar to the aerosol scenarios, the total economic impacts of the transference release scenarios are similar to impacts of an aerosol release, including the distribution of impacts by production types across time. Losses to producers range from $19,660 million to $61,445 million while changes in consumer welfare are a positive $3,576 million to a negative $59,895. Government indemnification and non-indemnification costs range from $163 million and $27 million to $9,850 million and $5,621 million, respectively. Regional non-agricultural impacts range from $163 million to $4,087 million in the losses resulting from hypothetical FMD outbreaks.

#### Tornado Release

Tornado events are unlike the previous releases because of immediate, widespread dispersion and the events are effectively self-announced. The economic impacts were estimated in the event the FMDv is released because a tornado comprises the containment laboratories at NBAF. With a tornado release event, the duration of an outbreak under the P5/P5 scenario is 240 days compared to 0, 28, and 35 days in the liquid waste, aerosol, and transference scenarios, respectively. Additionally, the number of animals culled in the P5/P5 scenario is significantly higher when compared to the other three release scenarios (approximately 5.8 million more animals are culled). Thus, the lower end of the range of total economic impacts for this scenario is much larger because more animals are culled and the duration is longer at the lower end of the distribution (P5/P5). Although the impacts are larger at the lower end of the distribution, the impacts at the upper end of the distribution (P95/P95) are smaller because fewer animals are culled. The total economic impacts range from losses of $76,426 million to $137,565 million. Similar to the aerosol and transference release events, producers lose along the entire distribution of outcomes. However, consumers do not gain in any at any point along the distribution. When compared to the other three release events, the range of government costs is smaller. However, the P5/P5 scenario has much larger government costs while the P95/P95 costs are smaller for both indemnification and non-indemnification. The lower and upper ends of the distribution for regional non-agricultural impacts are losses of $760 million and $3,828 million, respectively.

## Conclusions and Implications

United States animal agriculture is becoming a highly integrated and global system that is very important both domestically and internationally. This complex system combined with the increasing frequency of emerging infectious disease threatens the stability of the U.S. economy, food security, and livestock and public health. This economic analysis is part of a congressionally mandated site specific risk assessment, which links outcomes from plume and epidemiological models to risk outcomes. This study examines the economic consequences of foot-and-mouth disease virus (FMDv) releases from a national disease research facility. Specifically, we investigate the economic impacts to consumers and firms, costs to the government, and disruptions to non-agricultural regional sectors.

Outcomes of the site-specific risk assessment have been and are currently being implemented in the form of feedback into the planning and construction process of the National Bio- and Agro Defense Facility (NBAF), along with improvements in scientific and economic modeling. Given the projected investment costs of over $1 billion and the risks related to potential foreign animal and zoonotic disease releases, feedback into the planning and construction of the facility is critical to enhance future food security.

Unlike previous studies that focused on various alternate mitigation strategies, this study focuses on potential animal disease releases from NBAF. The release events modeled include aerosol, liquid waste, and transference (unannounced release events) and a tornado (announced release event). Indeed, differences in the distribution of economic consequences arise between the unannounced and announced events. Mitigation controls used includes stamping out, vaccinate-to-live and vaccinate-to-kill. Although this is not a definitive study of FMD vaccination, the economic consequences from the selected scenarios are informative to government planners and policy makers. Schroeder et al. [3] find that emergency vaccination can be a cost effective mitigation tool that can help reduce the spread of disease. They conclude that a high-capacity emergency vaccination program together with large vaccination zones results in significant savings to consumers, producers, and the government.

Total losses for the reported FMDv release events range from about $16 billion to $140 billion in damages. Producer effects are always negative due to lost output and reduced prices and share the largest burden in losses. Consumers realize negative or positive effects primarily contingent upon the size of the outbreak, export losses, and assumed demand shocks. Regional non-agricultural losses and government indemnification (non-indemnification) costs are much smaller than the producer and consumer impacts, ranging from $39 million to over $4,500 million and from $18 million to nearly $10,000 million ($3 million to over $5,600 million) across the scenarios, respectively. These economic impacts across the four release events are similar, except for the tornado release event. Because the tornado release event is effectively an announced event and the virus dispersion is greater, lower bound economic losses are much larger than the unannounced releases while the upper bound of the economic consequences is much smaller.

Several additional key insights are identified. First, it is important to integrate time into the economic analysis, as livestock are durable goods. Costs and benefits evolve over time in response to producer decisions, consumer reactions, and international trade responses by trading partners. Second, disaggregation among production types is vital to link epidemiological to economic models. Furthermore, this allows for additional analysis on how the different production sectors are impacted. Third, size of the outbreak and duration of trade sanctions are important. Fourth, the timing of identifying an FMDv release (announced vs. unannounced) is important and has differing economic consequences. Similar to all hypothetical foreign animal disease studies that report economic consequences, they are conditional in nature. Not only are they conditional on the available information and modeling assumptions, but they are conditional on an outbreak occurring. While it is plausible that FMDv releases may arise from NBAF, estimated probabilities of the sequence of events leading to an outbreak are not large [16]. Nevertheless, as actual events and empirical evidence demonstrate, FMD events are low probability high cost events that deserve continued vigilance and research to mitigate the consequences.

This study is not without limitations. Total costs could be overestimated if, for example, the impact of tourism is diverted to different areas or the purchase delayed/deferred. However, the estimated costs provide plausible estimates using standard economic techniques. The modeling approach assumes homogenous commodities and goods, including beef. FMD outbreaks are treated as shocks to economic system and are not endogenous to the model. Trade is not differentiated among trading partners, but rather differentiated by domestic and international markets. Capacity constraints need to be investigated for processing infected animals, as well as capital constraints. Nevertheless, the modeling framework provides the appropriate fidelity to assess economic consequences.

This study does raise questions for future research. First, additional research needs to be completed on vaccination strategies and capacity constraints. Second, trade sanctions and trade agreements need to be studied further. Third, government costs and the structure of government costs should be examined to better understand the nature of public expenditures. Fourth, potential benefits resulting from research, diagnostic tests, and training of personnel would have positive impacts. The extent of those benefits needs to be investigated. Fifth, sensitivity analysis on the economic parameters deserves further examination. Finally, further implementation of an iterative risk assessment may provide interactive feedback and subsequent updating of information that could increase the efficiency and effectiveness of the risk assessment and facility construction process.
